# Obesity and associated type 2 diabetes and hypertension in factory workers of Bangladesh

**DOI:** 10.1186/s13104-015-1377-4

**Published:** 2015-09-19

**Authors:** Bishwajit Bhowmik, Faria Afsana, Tareen Ahmed, Sadeka Akhter, Hasan Ali Choudhury, Anisur Rahman, Tofail Ahmed, Hajera Mahtab, A. K. Azad Khan

**Affiliations:** Executive Diabetes care Centre, Diabetic Association of Bangladesh, Dhaka, Bangladesh; Department of Endocrinology, Bangladesh Institutes of Research and Rehabilitation of Diabetes, Endocrine and Metabolic Disorders (BIRDEM), Dhaka, Bangladesh; Diabetes Prevention Program in Work Places, Diabetic Association of Bangladesh, Dhaka, Bangladesh

**Keywords:** Obesity, Type 2 diabetes, Hypertension, Factory worker, Bangladesh

## Abstract

**Background:**

Recent data suggest that the prevalence of obesity and its associate cardiometabolic risks are increasing in Bangladesh. Published data of obesity in Bangladeshi industry workers is scarce. The purpose of this study was to assess the prevalence of general and central obesity in Bangladeshi factory workers and their associations with diabetes and hypertension.

**Methods:**

A total of 791 male factory workers aged ≥20 years in capital Dhaka city of Bangladesh were investigated in a population-based cross-sectional survey. According to the International Association for the Study of Obesity and the International Obesity Task Force guidelines for Asian population, general obesity was defined as body mass index (BMI) ≥25 kg/m^2^, central obesity was defined as a waist circumference (WC) of ≥90 cm and waist hip ratio (WHR) of ≥0.90. Pearson’s correlation coefficient and logistic regression analysis were used to observe the association between anthropometric indices (BMI, WC and WHR) and cardiometabolic risk indicators (FBG, 2hBG, SBP and DBP).

**Results:**

The prevalence of overweight (BMI 23–24.9 kg/m^2^) and general obesity (BMI ≥25 kg/m^2^) in this study population was 29.8 and 43.5 % respectively. Central obesity defined by WC and WHR was 35.3 and 78.3 % respectively. Both general and central obesity were found to be significantly associated with diabetes and hypertension in separate logistic regression analyses.

**Conclusion:**

The prevalence of general and central obesity in Bangladeshi factory workers was high, and it was associated with diabetes and hypertension.

## Background

The prevalence of obesity is increasing at an alarming rate worldwide [1, 2]. Epidemiologic studies indicate that obesity is a major risk factor for cardiovascular disease, type 2 diabetes (DM), hypertension (HTN), cancer and premature death [2]. The high prevalence of obesity, combined with its concomitant cardiometabolic risks, makes it a global health challenge. Obesity is also increasing in Bangladesh. In 2010, World Health Organization (WHO) Risk Factor Survey estimated the prevalence of general obesity [Body mass index (BMI) ≥25 kg/m^2^] in people over 15 was 11 % and central obesity [waist circumference (WC): ≥94 cm in men and ≥80 cm in women] was 14 % in Bangladesh [3]. Bangladesh Demographic and Health Survey (BDHS) 2011 reported 17 % overweight or obesity (BMI ≥25 kg/m^2^) in adult Bangladeshi population [4]. However, these studies used WHO cut-off levels for western population. A rural study assessed the prevalence of general (BMI  ≥25 kg/m^2^) and central obesity (WC: ≥90 cm in men and ≥80 cm in women) between 1999 and 2009 and reported increase trends of prevalence. Prevalence of general obesity increased from 4.6 to 27.3 % and central obesity increased from 5.4 to 48.2 % in 10 years [5]. Most **i**mportantly, studies in rural Bangladesh have also found a significant association between obesity and major cardiometabolic risk factors such as diabetes and hypertension [6]. These findings indicate the need of more information from different socio-demographic areas of our country for the development of public health strategies for its primary prevention and treatment.

Although Bangladesh is an agro-based country its industrial sector has shown a marked improvement over the last few decades. The industrial sector now contributes around 28.6 % of the gross domestic product (GDP) but healthcare facilities for workers and employees are still inadequate in most of the industries in Bangladesh [7]. Studies conducted in USA [8], Australia [9], Italy [10] and India [11] have found increasing prevalence of obesity and other chronic diseases including diabetes, coronary artery disease and hypertension in industrial workers. Chronic diseases make those workers less productive, more prone to injury and have higher claim costs [8–10]. Therefore, if strategies are not developed to address this issue, there could be significant consequences for both employers and employees.

Considering the high prevalence of obesity and its relationship with diabetes and hypertension in our country, the “Diabetes Prevention Program in Work Places (DPWP)” study was conducted to assess the prevalence of general and central obesity in Bangladeshi factory workers and their associations with diabetes and hypertension.

## Methods

The participants of this study were taken from the Diabetes Prevention Program in Work Places (DPWP), an ongoing diabetes prevention program of Diabetic Association of Bangladesh (BADAS). The main objective of this program is to establish a model surveillance system with special focus on diabetes for industry workers in Bangladesh. Program consists of screening of diabetes at work places and then organize education sessions about healthy lifestyle for all the participants. Individuals diagnosed with diabetes are referred to nearby BADAS centers for registration, treatment and further follow-ups. Furthermore, diagnosed prediabetes (impaired fasting glucose and or impaired glucose tolerance) individuals are invited for participate in diabetes prevention program of BADAS. In this study individuals were randomly selected from five industries (two pharmaceuticals, one tobacco, and two footwear industries) have already been enlisted in DPWP. To determine the required sample size, the formula: n = z^2^PQ/d^2^ was used, where P stands for prevalence (diabetes plus prediabetes) from a previous study [12], i.e. 0.165 (16.5 %); Q = 1 − P, i.e. 0.835 and d = allowable margin of error of known prevalence i.e. 0.025 (2.5 %). Thus the calculated sample size was, n = 850. One thousand individuals aged ≥20 years of those 5 industries were invited to participate in this study by following simple random procedure. Of these, 870 (male 800 and female 70) agreed to participate. Response rate was 87 %. It should be noted that male are still dominating the labor market in Bangladesh except garments and textile industries. The study was conducted in between January 2011 to November 2013. The inclusion criteria were: ≥20 years of age, have permanent job for more than 5 years, willing to participate, and being able to communicate. The exclusion criteria were: acute or chronic cardiac, renal or hepatic illness, and pregnancy at the time of screening. All potential participants received a letter inviting them to participate in the study. Written consent was obtained from each individual prior to inclusion. Study participants were also verbally informed of their right to withdraw from the study at any stage, or to omit their data from the analysis. In addition participants received a written copy of their rights during and following the completion of the study. The study protocol was approved by ethical review committee of Diabetic Association of Bangladesh for medical research.

Survey procedures included completing a questionnaire on socio-demographic information, anthropometric measurement, blood pressure measurement and measurement of both fasting blood glucose (FBG), and oral glucose tolerance test (OGTT). Anthropometric measurements, such as height, weight and waist and hip circumferences, were taken with the participants wearing light clothes and without shoes. BMI was calculated as the weight (kg) divided by square of the height (m^2^). Waist circumference (WC) was measured by placing a tape horizontally midway between the lower margin of the last palpable rib and iliac crest on the mid-axillary line. Hip circumference was measured at a level parallel to the floor, at the largest circumference of the buttocks. Waist hip ratio (WHR) was then calculated from waist and hip circumference (cm). Blood pressure was measured in sitting position by aneroid sphygmomanometer on right hand, after a 10 min rest, and with standard procedure. A second reading was taken after 2 min resting interval. Mean of two measurements of blood pressure was used in the statistical analysis. Both fasting and 2-h blood glucose were measured using the portable glucometer (One Touch II, Lifescan, Milpitas, CA, USA) in whole blood obtained by finger prick in the middle finger—an approach that is widely used in resource-limited countries [4]. Blood glucose measurements were adjusted to obtain equivalent plasma glucose levels [13].

According to the International Association for the Study of Obesity and the International Obesity Task Force guidelines, overweight was defined as a BMI of 23.0–24.9 kg/m^2^, while obesity was defined as BMI ≥25 kg/m^2^ [14, 15], central obesity was defined as a WC of ≥90 cm and waist hip ratio (WHR) of ≥0.90. Diabetes was diagnosed if FBG value was ≥7.0 mmol/l and/or 2-h post blood glucose was ≥11.1 mmol/l [16], self-reported T2DM, or use of diabetes medication. Hypertension (HTN) was defined as a systolic blood pressure (SBP) of ≥140 mmHg and/or diastolic blood pressure (DBP) of ≥90 mmHg [17] or if they were receiving treatment for HTN. Based on the monthly expenditure socio-economic condition was classified as low [<6000 Bangladeshi Taka (BDT, 1 USD = 84 BDT)], medium (6000–11,000 BDT) and high (>11,000 BDT). Education level was graded as illiterate: unable to read and write; undergraduate: having primary (from grades 1 to 5), secondary (from grades 6 to 10) and higher secondary education (from grades 11 to 12); graduate. Physical activity was graded on the ordinal scale of 1–3, corresponding to light, moderate and heavy, according to the activity level based on their occupation. For the purpose of data analysis, these results were transformed into a binary variable—inactive (grade 1) and active (grade 2 and 3).

### Statistical analysis

Due to limited number female participants (n = 70), only 800 male data were considered for the analysis. The present analysis is based on 791 male for whom all the variables were available. Means and percentages with 95 % confidence intervals adjusted for age were given for normally distributed continuous variables and categorical variables as needed. Age standardized prevalence by direct standardization method were estimated on the basis of 2001 census data before performing statistical tests [18]. Differences between the groups of means and proportions adjusted for age were tested by analysis of covariance (ANCOVA) and logistic regression. Measures of association between anthropometric indices (BMI, WC and WHR) and cardiometabolic variables (SBP, DBP, FBG and 2hBG) were tested by Pearson’s correlation coefficient. In logistic regression analyses controlling by age, was assessed between (a) general obesity and (b) central obesity (WC and WHR) as categorical independent variables and, taken one by one, diabetes and hypertension as dependent variables. Both STATA 11 for Windows (STATA Co., College Station, TX, USA) and PASW statistics version 21 for Windows (SPSS Inc., Chicago, IL, USA) were used as needed. A p value <0.05 was considered statistically significant.

## Results

Basic characteristics of the study population are shown in Table 1. Among the total number of participants, the mean age and weight of the study population was 38.3 years and 68.2 kg respectively. Nineteen percent (19.0 %) of study participants were of high socio-economic status, 18.8 % had higher education and 35.9 % were physically inactive. Among the participants, 38.9 and 15.9 % had family history diabetes and hypertension and 15.4 and 27.9 % had diabetes and hypertension respectively.

**Table 1:** Basic characteristics of the study population (n = 791)

Variable	Mean/percentage (95 % CI)
Age (year)^a^	38.9 (38.3, 39.6)
Weight in kg^a^	68.2 (67.5, 68.9)
High socio-economic status^b^	19.0 (16.3, 21.7)
Higher education^b^	18.8 (16.1, 21.4)
Physical inactivity^b^	35.9 (32.6, 39.1)
Family history of diabetes^b^	38.9 (35.5, 42.3)
Family history of hypertension^b^	15.9 (13.5, 18.6)
Diabetes^b^	15.4 (12.9, 17.9)
Hypertension^b^	27.9 (24.8, 31.1)

^a^Values are presented as mean with 95 % confidence interval

^b^Values are presented as percentage (%) with 95 % confidence interval

Mean BMI and the prevalence of overweight and obesity by age are shown in Table 2. Age adjusted mean of overall BMI was 24.5 kg/m^2^. The age standardized prevalence of overweight and obese were 29.8, and 43.7 %, respectively.

**Table 2:** Mean body mass index (BMI) and the prevalence of overweight (23–24.9 kg/m^2^) and obesity (≥25 kg/m^2^) by age

Age group (years)	Number	BMI	Overweight	Obese
		Mean (95 % CI)	% (95 % CI)	% (95 % CI)
Men				
20–30	178	23.6 (23.1, 24.1)	27.5 (20.9, 34.1)	30.9 (24.1, 37.7)
31–40	246	25.2 (24.8, 25.6)	28.5 (22.8, 34.1)	52.0 (45.8, 58.3)
41–50	292	25.0 (24.7, 25.4)	32.2 (26.8, 37.6)	45.5 (39.8, 52.3)
≥51	75	24.8 (23.9, 25.6)	30.7 (20.1, 41.2)	40.0 (28.8, 51.1)
Age adjusted	791	24.5 (22.2, 22.4)	29.8 (26.7, 33.0)	43.7 (40.3, 47.2)

Values are presented as mean (95 % confidence interval) or % (95 % confidence interval) adjusted for age as indicated

*CI* confidence interval

Mean of WC and WHR and the prevalence of central obesity by age are shown in Table 3. The age adjusted mean of central obesity based on WC and WHR were 86.6 cm and 0.93 respectively. There was no significant mean difference observed between the age groups. The age standardized prevalence of central obesity based on WC and WHR were 35.3 and 78.3 % respectively.

**Table 3:** Mean of waist (WC) and waist hip ratio (WHR) and the prevalence of central obesity by age

Age group (years)	WC			WHR		
	Number	Mean (95 % CI)	Central obesity by WC (95 % CI)	Number	Mean (95 % CI)	Central obesity by WHR (95 % CI)
Men						
20–30	178	85.1 (82.8, 87.3)	24.9 (12.1, 37.7)	178	0.91 (0.89, 0.93)	78.6 (70.5, 86.8)
31–40	246	88.0 (86.9, 89.2)	32.9 (25.7, 40.1)	246	0.94 (0.93, 0.94)	83.3 (78.9, 87.8)
41–50	292	87.5 (86.2, 88.9)	42.6 (33.9, 51.2)	292	0.94 (0.93, 0.95)	73.1 (60.2, 85.9)
≥51	75	85.8 (82.7, 88.9)	34.9 (16.4, 53.6)	75	0.93 (0.91, 0.95)	45.7 (22.1, 69.2)
Age adjusted	791	86.6 (85.9, 87.3)	35.3 (32.0, 38.5)	791	0.93 (0.92, 0.94)	78.3 (75.4, 81.2)

Central obesity by WC: (≥90 cm); central obesity by WHR: (≥0.90). Values are presented as mean (confidence interval) or % (95 % confidence interval) adjusted for age as indicated

*CI* confidence interval

Cardiometabolic risk factors in both general and central obesity by WC and WHR are shown in Table 4. All the cardiometabolic risk parameters including blood pressure and glycemic parameters were comparable between all three groups. Higher rate of DM was observed in both general and central obesity groups and higher rate of HTN was only observed in central (define by both WC and WHR) obesity groups.

**Table 4:** Differences in cardiometabolic risk factors based on general obesity, central obesity by WC and central obesity by WHR

Cardiometabolic factor	General obesity by BMI		Central obesity by WC		Central obesity by WHR	
	Non-obese <25 kg/m^2^	Obese ≥25 kg/m^2^	Non-obese <90 cm	Obese ≥90 cm	Non-obese <0.90	Obese ≥0.90
SBP (mmHg)	117.3 (115.8, 118.7)	121.9 (120.3, 123.4)*	118.3 (116.9, 119.6)	122.3 (120.6, 124.0)*	114.9 (112.4, 117.5)	120.3 (119.0, 121.6)*
DBP (mmHg)	78.8 (77.9, 79.6)	81.7 (80.8, 82.6)*	79.1 (78.4, 79.9)	82.2 (81.1, 83.2)*	76.9 (75.4, 78.4)	80.5 (80.1, 81.6)*
HTN, %	25.4 (21.5, 29.2)	29.7 (25.0, 34.5)	24.5 (20.8, 28.1)	33.4 (27.9, 38.8)*	19.2 (12.4, 26.1)	30.2 (26.3, 34.1)*
FBG (mmol/L)	5.8 (5.7, 5.9)	6.0 (5.8, 6.2)*	5.8 (5.7, 5.9)	6.1 (5.9, 6.3)*	5.6 (5.4, 5.9)	6.1 (6.0, 6.3)*
2hBG (mmol/L)	7.8 (7.5, 8.0)	8.0 (7.7, 8.4)	7.7 (7.4, 8.0)	8.3 (7.9, 8.7)*	7.2 (6.7, 7.7)	8.4 (8.1, 8.7)*
DM, %	11.6 (8.7, 14.4)	19.2 (15.1, 23.2S)*	10.7 (8.0, 13.4)	22.6 (178, 27.4)*	5.5 (1.5, 9.5)	20.3 (16.8, 23.9)*

Values are presented as mean (95 % confidence interval) or % (95 % confidence interval) adjusted for age as indicated. P < 0.05

* P < 0.05 between non-obese and obese in the same group

*BMI* body mass index, *WC* waist circumference, *WHR* waist hip ratio, *SBP* systolic blood pressure, *DBP* diastolic blood pressure, *HTN* hypertension, *FPG* fasting plasma glucose, *2hPG* 2 h plasma glucose

Correlation between BMI, WC and WHR and cardiometabolic risk factors are presented in Table 5. A significant positive correlation was seen in between BMI, WC and WHR with SBP, DBP, FBG and 2hBG.

**Table 5:** Correlation (r) analysis of body mass index (BMI), waist circumference (WC) and waist hip ratio (WHR) with cardiometabolic risk factors

Variables	BMI		WC		WHR	
	r	P value	r	P value	r	P value
Men						
SBP	0.217	<0.001	0.254	<0.001	0.214	<0.001
DBP	0.238	<0.001	0.296	<0.001	0.231	<0.001
FBG	0.197	<0.001	0.207	<0.001	0.270	<0.001
2hBG	0.134	0.001	0.171	<0.001	0.287	<0.001

*SBP* systolic blood pressure, *DBP* diastolic blood pressure, *FPG* fasting plasma glucose, *2hPG* 2 h plasma glucose

Table 6 shows the odds ratio of DM and HTN for general obesity by BMI and central obesity by WC and WHR after adjusted for age. WC and WHR were significantly associated with both DM and HTN. However, BMI was only significantly associated with DM only. WHR was the most sensitive index for diabetes (OR 3.90 p < 0.001) and HTN (OR 1.80 for male p < 0.021).

**Table 6:** Adjusted odds ratio (OR) of diabetes (DM) and hypertension (HTN) for general obesity (BMI ≥25 kg/m^2^), central obesity by WC (≥90 cm) and central obesity by WHR (≥0.90)

	General obesity by BMI		Central Obesity by WC		Central Obesity by WHR	
	OR (95 % CI)	P value	OR (95 % CI)	P value	OR (95 % CI)	P value
Men						
DM	1.70 (1.14, 2.52)	0.008	2.39 (1.61, 3.57)	<0.001	3.90 (1.76, 8.73)	0.001
HTN	1.18 (0.86, 1.62)	0.304	1.54 (1.11, 2.14)	0.009	1.80 (1.10, 2.99)	0.021

Adjusted odds ratio after logistic regression analysis adjusted for age

*BMI* body mass index, *WC* waist circumference, *WHR* waist hip ratio, *CI* confidence interval

## Discussion

This is the first population-based study to explore the prevalence of general and central obesity among factory workers in Bangladesh and their association with DM and HTN. In this study, mean BMI was 24.5 g/m^2^ and age standardized prevalence of overweight and obesity were 35.3 and 78.3 % respectively. The findings in this study were higher from the previous studies conducted in both urban and rural Bangladeshi population [6, 19, 20]. Our study findings were also higher than studies conducted in Indian factory workers [11, 21]. However, they used different cut-off levels to define overweight and obesity. In addition, place of study and study participants were also different from our study participants. Age adjusted mean based on WC and WHR were 86.6 cm and 0.93 and age standardized prevalence of central obesity based on WC and WHR were 35.3, and 78.3 % respectively for the study population. The findings of the study were consistent with the studies conducted in both northern and Southern Indian workers [11, 21].

The prevalence of central obesity was higher than general obesity in our study which indicate a significant portion of the population may not be classified as obese by BMI levels. These study findings are consistent with previous rural Bangladeshi study [6]. Hence, it has been suggested that a single BMI cut-off level for both male and female might not be adequate to define general obesity. Sex and ethnic specific BMI levels for defining general obesity might be preferable. In our study, the highest prevalence of both general and central obesity was observed in the middle-aged (30–50 years) group which is consistent with our previous rural studies [5, 6, 12]. Age related change, i.e., secretion of male hormone testosterone could be the underline cause of our finding. Higher rate of DM and HTN was observed in both general and central obesity groups in our study. Prevalence of these risk factors increased during the most productive years putting them at risk of cardiovascular morbidity and mortality at relatively younger age.

We have found a significant but weak association (correlation) between obesity indices (BMI, WC and WHR) and cardiometabolic risk factors including SBP, DBP, FBG, and 2hBG. However, significant associations were identified for the dichotomized variable (odds ratios). Size of the sample and underline confounding effects could be the key factors but need more in-depth study including all the possible influences to confirm the above mentioned findings.

In our study, we have found that the indices of central obesity (WC and WHR) were more closely associated with DM than general obesity (BMI). However, no significant difference were observed between general and central obesity indices for predicting HTN. In the present study, WHR was the most sensitive risk indicator of DM and HTN which is in agreement with studies conducted in different Asian and as well as in Bangladeshi populations [6, 22–25]. From a pathophysiological point of view, central obesity has been found to play a vital role in the pathogenesis of insulin resistance, which is a key factor leading to the development of DM and HTN.

Limitations of the study are that this was a cross-sectional study. Therefore, the association between diabetes, HTN and obesity cannot be clearly established. As the study was carried out in an industrial setting, it may not be representative of the general population. However, this study is broadly generalizable to similar large, organized sector workforce in Bangladesh. Further, our study population comprised only men. The results of this study cannot be generalized to women, in whom the risk factor as well as disease burden could be different than that reported in this study population. Our study has several strengths including high response rate (87 %), followed by simple random procedure for sample collection, and use of highly trained interviewers to ensure the quality of the data collection.

## Conclusion

Our study findings documented that the prevalence of both general and central obesity in adult Bangladeshi factory workers is high, and both the obesity indices were associated with DM and HTN. So the high prevalence of non-communicable disease risk factors including obesity, DM and HTN in Bangladeshi industrial settings as a cause of concern as well as an opportunity for carrying out work place interventions. This industry workers provided an opportunity to study the influence of socioeconomic and lifestyle transition on the prevalence of obesity and associated DM and HTN. Furthermore, we need well design longitudinal studies to confirm our findings. Our results support the need for periodic low-cost screening programs as well as awareness program about healthy lifestyle for the employees. In addition, we also recommended periodic follow-ups of the employees with DM and HTN.
